# Novel epoxy-bPBD-BisMSB composite plastic scintillator for alpha, beta and gamma radiation detection

**DOI:** 10.1038/s41598-023-45501-9

**Published:** 2024-03-19

**Authors:** R. M. Sahani, Arun Pandya

**Affiliations:** Nuclear Radiation Management and Application Division, Defence Laboratory (DRDO), Jodhpur, 342011 India

**Keywords:** Nuclear physics, Particle physics

## Abstract

A composite plastic scintillator is prepared by uniform dispersion of organic fluorophores 2-(4-Biphenylyl)-5-(4-tert-butylphenyl)-1,3,4-oxadiazole (b-PBD) and 1,4-bis(2-methylstyryl) benzene (Bis-MSB) in epoxy resin followed by curing at room temperature. The developed scintillator is strong blue emitter (425 nm), confirmed by 365 nm UV excited Photo luminescence and beta particle (^90^Sr-^90^Y) excited Radio-luminescence characterizations. The developed scintillator is highly transparent (~ 70%) to emitted light wavelength. Moreover, the scintillator’s blue emission is appropriate for photomultiplier tube (PMT) based scintillation measurement due to its maximum peak spectral response in blue region. Alpha, beta and gamma radiation detection were performed on PMT coupled scintillators of sizes Ø50 mm × 1 mm, Ø50 mm × 5 mm and Ø50 mm × 25 mm respectively. Pulse height spectra were recorded using 1 k Multichannel analyser (MCA) using various reference radiation sources. All scintillators demonstrated promising response to the respective radiations. Absolute detection efficiency of alpha scintillator is obtained as 32% (^241^Am), 86% of that of standard plastic scintillator EJ-212. Beta endpoint energy and gamma Compton edges showed linear variation w.r.t. corresponding channel numbers. Detection efficiency of beta and gamma scintillator is found to be 35.7% (^90^Sr-^90^Y) and 6.7% (^136^Cs) respectively. The developed scintillator has potential to be used for radioactivity contamination & gamma dose rate measurement applications.

## Introduction

Scintillators are energy converting luminescent materials that emit ultraviolet or visible light pulses when excited by high energy particles (alpha & beta particle, protons or neutrons) or high energy photons (X-ray and Gamma radiation). A scintillation detector is realized by coupling it to a photo-detector that produces electrical pulses on absorption of these light pulses, which can be processed and correlated with incident radiation intensity and energy^1^. Scintillation detectors have various applications such as radiation spectrometry^2^, medical imaging, positron emission tomography(PET)^3^, Industrial radiography^4^, high energy physics experiments^5^, radioisotope identification^6^, homeland security^7^ and nuclear & radiological emergency management^8^. Scintillators are broadly categorized in two types (i) Inorganic scintillators and Organic scintillators. Inorganic scintillators are characterized by high stopping power due their high density (3.3 to 9.4 g/cc), high light yield and good energy resolution^9,10^. Single crystals^11^, transparent ceramics^12^, composites^13,14^ and glasses^15^ are the various forms of inorganic scintillators. Various inorganic single crystals of halides, oxides, silicates, aluminates and germinate single crystals are being used for radiation detection and measurement application among which NaI:Tl, CsI:Tl, LaBr3:Ce, BGO, CLYC:Ce are popular scintillators^16^. GYGG:Ce transparent ceramic scintillator has been emerged as alternative to single crystal due to its ease of fabrication and cost effectiveness of process^17^. Inorganic Ce doped silicate glass scintillator along with ^6^Li are used for thermal neutron detection in high temperature environment^18,19^.

Organic Scintillators consist of fluorescent aromatic molecules and are characterized by low density (1.03–1.2 g/cc), fast decay times (0.7–3.3 ns)^20^ and excellent pulse shape discrimination (PSD) capability making it highly suitable for gamma-neutron discrimination^21^. These scintillators can be fabricated as single crystals, liquid scintillator or plastic scintillator. Naphthalene, stilbene, anthracene are important organic single crystal scintillators. Organic liquid scintillator is solution of fluorophores such as p-terphnyl, PPO etc., in organic solvents such as benzene, xylene etc., used for low energy beta measurement^22^. Plastic scintillator is a solid scintillator having transparent polymer as base with primary and secondary fluorophores dissolved in it. The polymer base primarily absorbs radiation energy and subsequently transfers it to primary fluorophore molecules having emission UV range. The emitted UV light wavelength is shifted to visible range by a secondary fluorophore as wavelength shifter^23^ for making it suitable for PMTs. The advantage of plastic scintillator lies in its flexibility to cast in different shapes and sizes for gross counting^24^ and radioactive contamination monitoring applications^25,26^. Plastic scintillators have been modified by using multiple combinations of polymers and fluorophores as per the applications. Various plastic scintillators have been developed utilizing different polymer bases such as polyvinyl toluene (PVT)^27^, polystyrene (PS)^28^, Polymethyl Methacrylate (PMMA)^29^, poly(9-vinylcarbazole) (PVK)^30^, Polysixloxane^31^, Polyethylene Naphthalate (PEN)^32^ and Others. Generally, plastic scintillator is fabricated by starting from a monomer in which the fluorophores are dissolved and subjected to heat cycles for polymerization to obtain solid scintillator. This process of developing plastic scintillator is time consuming and may take several days. Among resin systems, epoxy resins are transparent in nature and it can be easily cured at room temperature conditions, significantly reducing working time and temperatures requirements. Further epoxy based scintillator are more resistant to temperature and chemicals and can be casted easily in multiple forms^33^.

In this paper, development of epoxy based composite plastic scintillator consisting of Butyl-PBD (bPBD) as primary fluorophore (λ_em_ ~ 364 nm) and Bis-MSB as wavelength shifter or secondary fluorophore (λ_em_ ~ 425 nm)^34^ is presented. The developed scintillators of sizes Ø50mm × 1 mm, Ø50 mm × 5 mm and Ø50 mm × 25 mm showed promising response to alpha, beta and gamma radiation respectively.

## Material and methods

### Preparation of epoxy composite plastic scintillator

**Figure 1:**
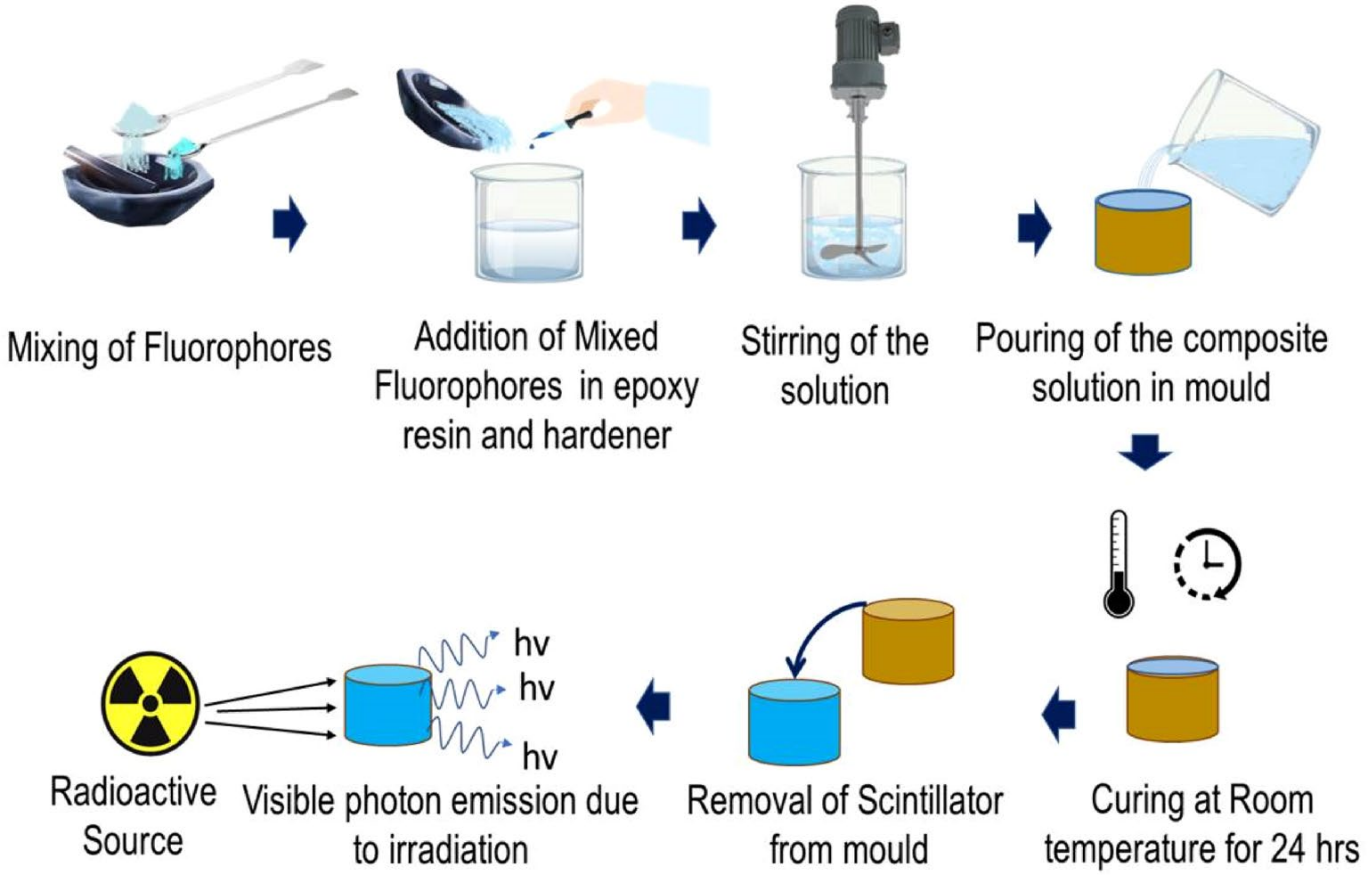
Schematic representation of step wise preparation process of epoxy-bPBD-Bis MSB composite plastic scintillator.

Fluorophores 2-(4-Biphenylyl)-5-(4-tert-butylphenyl)-1,3,4-oxadiazole (b-PBD) and 1,4-bis(2-methylstyryl) benzene (Bis-MSB) were taken in ratio 10:1, mixed and grinded using a mortar and pestle to obtain a uniform mixture. 1.1% w/w of fluorophore mixture was then added to epoxy resin in a plastic container and subsequently resin hardener was added. The solution was then stirred mechanically to disperse fluorophores uniformly in epoxy resin-hardener solution. After uniform dispersion of the fluorophores in the solution, it was then poured in Ø50 mm cylindrical moulds and for casting of 1 mm, 5 mm and 25 mm thick scintillators by controlling the volume of solution. The solution was left at room temperature for 24 h for curing to get solid composite plastic scintillators. Step wise preparation process is schematically shown in Fig. 1.

### Material characterization

Photoluminescence measurements were performed using JASCO 6000 Spectro-fluorometer utilizing 150W Xe-tube UV light source. Radio-luminescence was measured by replacing UV light by ^90^Sr-^90^Y (7.74 kBq) beta radiation source as excitation source. Transmittance was investigated by using Analytical Jena Specord-900 photodiode array spectrophotometer.

### Detector preparation

**Figure 2:**
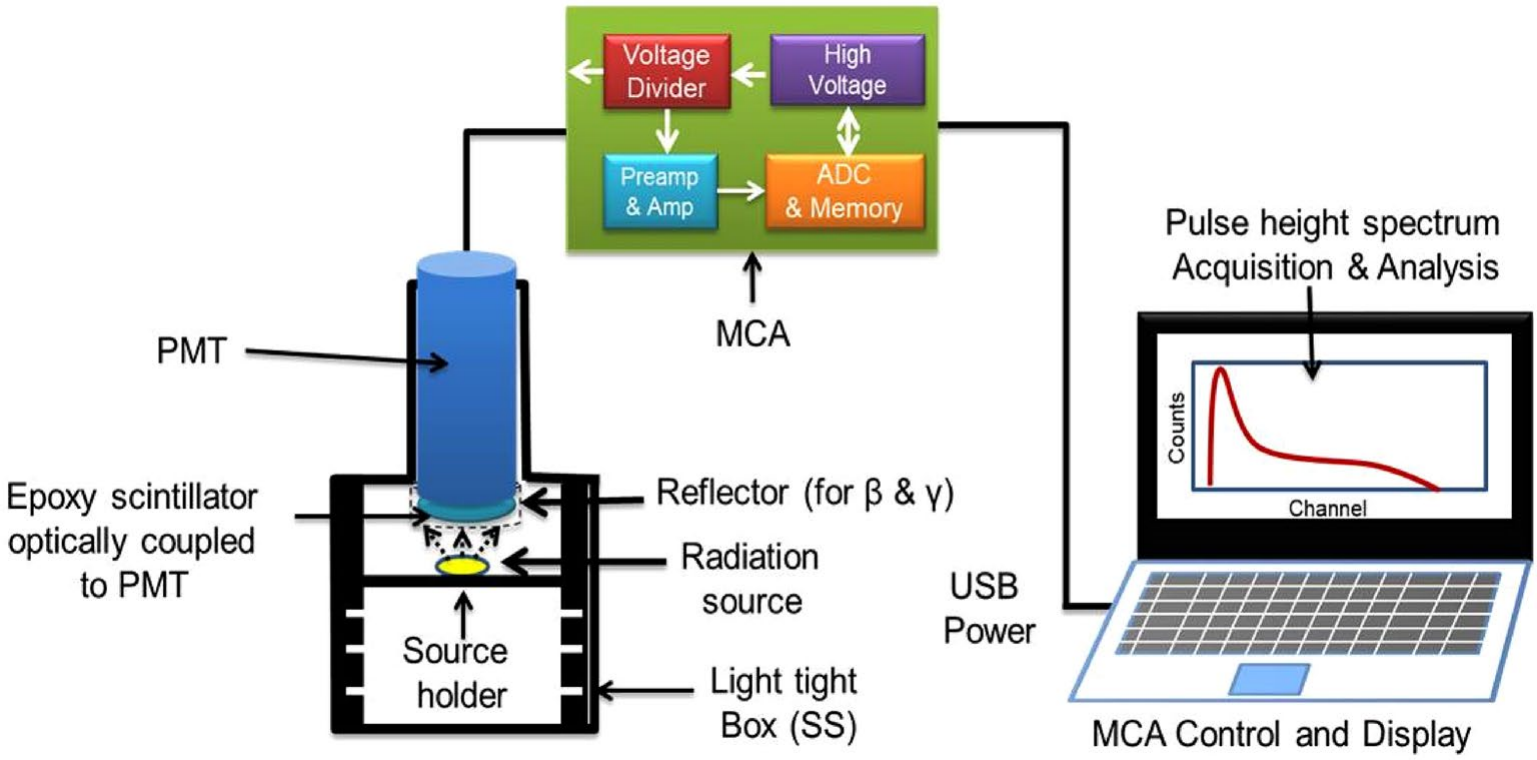
Schematic representation of experimental set-up used for alpha, beta and gamma radiation measurement using epoxy scintillator.

For detection of alpha, beta and gamma radiation, prepared scintillators of sizes Ø50mmx1mm, Ø50 mm × 5 mm and Ø50 mm × 25 mm were coupled to three Hamamatsu R-550 multi-alkali PMT using optical grade silicon grease to match the refractive index of PMT glass with epoxy composite plastic scintillator. An aluminium foil of thickness ~ 10 µm was wrapped on Beta and Gamma scintillators as reflector material for efficient collection of scintillation light. No aluminium foil is wrapped on alpha scintillator to avoid significant loss of alpha particle energy during its passage through the foil. Experimental setup used for detection of alpha, beta and gamma radiation is shown in Fig. 2 is quite similar to that of our earlier work^35^. Scintillator coupled PMT is connected to 1 k Multi channel analyser (MCA) (Make: Para Electronics, Model-GSpec-USB**)** for recording of radiation induced scintillation pulse height. MCA consists of inbuilt voltage divider of PMT, HV circuit, preamplifier and spectroscopy amplifier. The detector part was fitted in a stainless steel (SS) light tight box to prevent external light entrance during the measurement. The emitted scintillation photons, due to interaction of radiation with scintillator, were converted into electrical pulses by photo-multiplier tube. The generated pulses were further amplified by preamplifier & linear amplifier inbuilt in MCA. The amplified signals were converted to digital pulses by ADC and further plotted with respect to channel no. on a PC. The MCA was powered and controlled by the PC (Fig. 2).

### Radiation measurement

Reference radiation source of various activities and energies were used for radiation measurement experiments. For alpha radiation measurement ^241^Am + ^239^Pu (13 Bq), ^241^Am (3700 Bq) and ^239^Pu (22,200 Bq) reference sources were used. For beta radiation measurement ^147^Pm (1.495 kBq), ^60^Co (17.74 kBq), ^204^Tl (4.32 kBq) and ^90^Sr-^90^Y (5.09 kBq) were used and for gamma measurement, ^133^Ba (63.63 kBq), ^137^Cs(48.76 kBq), ^60^ Co (17.487 kBq) and ^22^Na (23.38 kBq) were used. Scintillation pulse height spectra using alpha, beta and gamma sources were recorded for duration of 300 s at closest source to detector distance (1 mm) and analysed. All measurements were performed in ambient conditions and no vacuum was used during the measurement. Ionizing radiations primarily interact with host polymer matrix (Epoxy) and deposit their energy by ionization and excitation of host polymer molecules. Alpha and beta particles being the charged particles, directly excite the host polymer molecules whereas gamma radiation being photon, indirectly deposit its energy by generating recoiled electrons through Compton scattering, which in-turn excites the host polymer molecules. The excitation energy is subsequently transferred to primary fluorophore Butyl-PBD (b-PBD), a broad UV emitter peaked at ~ 364 nm. Due to overlapping of excitation spectra of the wavelength shifter Bis MSB and emission spectra of b-PBD, Bis-MSB absorbs UV emission from b-PBD and coverts it into visible photons of 425 nm through fluorescence process^34^. These visible photons finally come out of the scintillator and reach to photocathode of PMT, generating photoelectrons which get multiplied in subsequent stages of dynodes before reaching to anode to constitute an electrical pulse. The electrical pulse in amplified and processed to get a pulse height distribution with help of MCA and displayed on PC.

## Results and discussions

**Figure 3:**
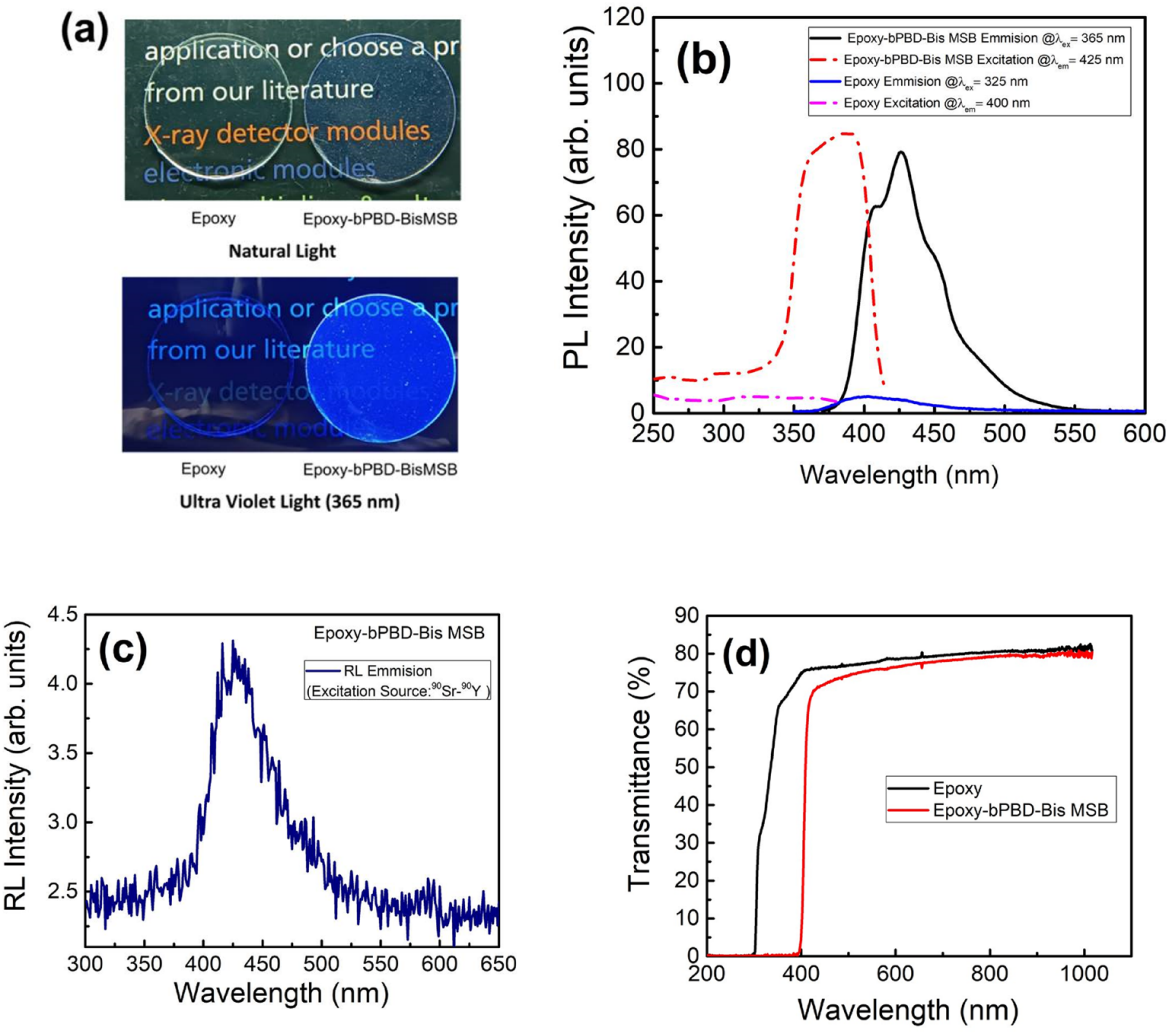
Optical and luminescence properties of developed scintillator (**a**) developed scintillator in natural and UV light showing strong blue emission in contrast with bare epoxy (**b**) excitation and emission photo-luminescence spectra of developed scintillator showing the emission peaking at ~ 425 nm suitable for photomultiplier tube based scintillation measurement. (**c**) ^90^Sr-^90^Y excited radio-luminescence luminescence spectra showing the emission ~ 425 nm (**d**) optical transmittance of developed composite scintillator in 200–1000 nm region showing the scintillator has ~ 70% transmittance to emitted wavelength (425 nm).

The developed scintillator along with bare epoxy is shown in Fig. 3a in natural light and UV lamp light (365 nm). It is evident that in natural light, developed scintillator is highly transparent and in contrast with bare epoxy, strong blue emission is clearly visible in UV light, confirming the luminescent nature of the scintillator. Photoluminescence excitation and emission spectra of developed scintillator and bare epoxy are shown in Fig. 3b. Broad emission of scintillator in visible range with peak at ~ 425 nm, obtained with 365 nm UV excitation, further substantiate the blue emission shown is Fig. 3a. Further, In order to be scintillator, the developed composite must show the property of radio-luminescence i.e. to emit visible light on excitation with ionizing radiation source. ^90^Sr-^90^Y (beta radiation source) is used to excite the developed plastic scintillator and Radio-luminescence (RL) generated is measured. The RL measurement also showed the emission peaked at ~ 425 nm, which confirms the developed scintillator is responding to ionizing radiation (Fig. 3c). Transmittance measurement showed that the scintillator is transparent to visible light and transmittance to RL emitted light wavelength (425 nm) is ~ 70% (Fig. 3d).

### Alpha, beta and gamma detection

#### Alpha radiation detection

**Figure 4:**
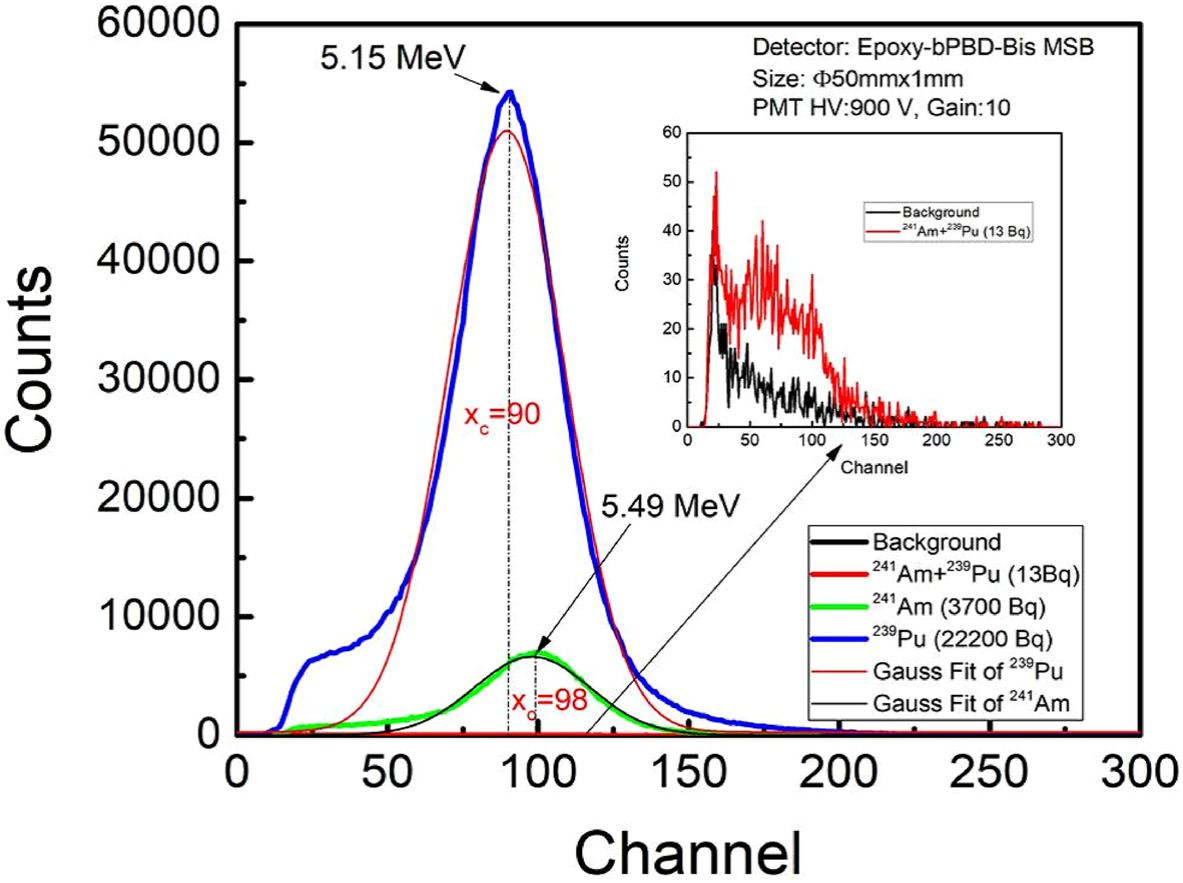
Pulse height spectra obtained from Ø50 mm × 1 mm composite plastic scintillator using various alpha radiation sources (^241^Am + ^239^Pu, ^241^Am and ^239^Pu). Background and response at low activity is shown in inset for clarity.

Figure 4 shows the pulse height spectra acquired for duration of 300 s with Ø50mmx1mm epoxy composite plastic scintillator using various activity alpha sources. Pulse height spectra obtained in absence of radiation source i.e. background is also shown for comparison. Pulse height intensities are found increasing with the activity of sources. Alpha detector is sensitive enough to distinguish the pulse height intensity obtained using low activity ^241^Am + ^239^Pu source (~ 13 Bq) in contrast of the background (shown in the inset). Mono-energetic alpha particles, deposit their energy in very short range and due to transparency of the scintillator the obtained alpha pulse height distribution is narrow compared to that of beta and gamma radiation. The pulse height distribution is nearly Gaussian. Peak centroids (x_c_) of pulse height spectra were obtained by Gaussian fit. ^239^Pu and ^241^Am spectra peak centroid were obtained at channel no. 90 and 98 which corresponds to alpha energies 5.15 and 5.49 MeV marked for each peak on on Fig. 4. The energies mentioned on Fig. 4 are standard energies of the sources. While passing through 1 mm of air between source and detector, 88.2 keV energy is lost which is 16% of initial energy^36^. The difference in peaks is attributed to dependence of total number photon generated due to deposited energy of alpha particles.

**Figure 5:**
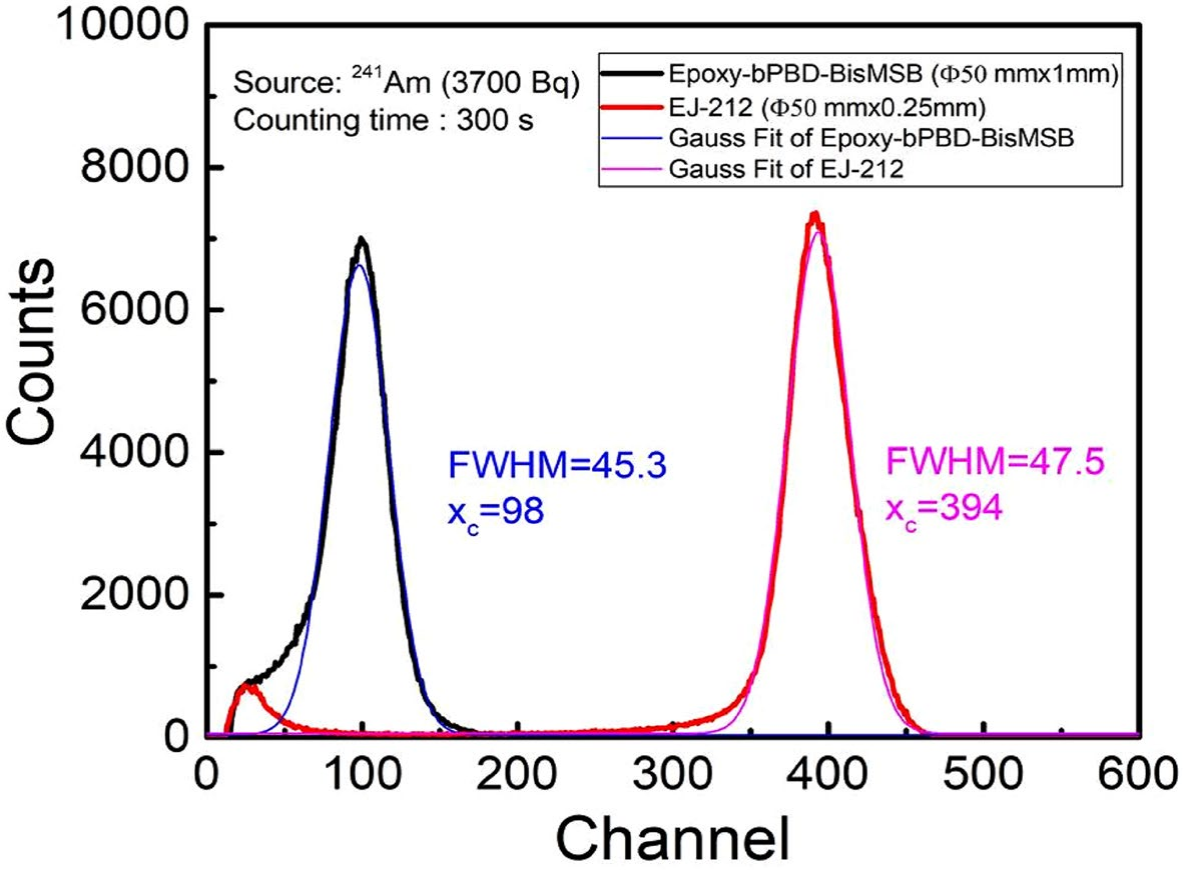
Alpha radiation response comparison of developed epoxy-bPBD-BisMSB (Ø50mmx1mm) composite scintillator with standard thin EJ-212 (Ø50 mm × 0.25 mm) plastic scintillator.

Alpha particle response of epoxy composite scintillator was compared with commercial thin plastic scintillator EJ-212. Figure 5 shows the pulse height spectra obtained using EJ-212 plastic scintillator (Ø50 mmx0.25 mm) along with pulse height spectra using Ø50 mmx1mm epoxy plastic scintillator shown for comparison. Peak centroid of spectra with EJ-212 was obtained at higher channel no. ~ 394 compared to that of epoxy scintillator (channel no. ~ 98). This is attributed to higher light yield (10,000 ph/MeV) of EJ-212, that results in higher pulse amplitude and lesser thickness allows the higher transmission of generated scintillation photons. The full width at half maximum (FWHM) obtained by Gaussian fitting, found to be ~ 45.3 for epoxy plastic scintillator which is comparable to FWHM ~ 47.5 of EJ-212 (Fig. 5). The absolute detection efficiency calculated from the expression

1 $$\varepsilon (\%)= \frac{{N}_{A}-{N}_{B}}{A}\times 100,$$

where ${N}_{A}$ is count rate in cps due to source of activity A and ${N}_{B}$ is background count rate in cps. Count rate is obtained by integrating area under the pulse height spectrum divided by counting time t. Absolute detection efficiency obtained for epoxy plastic scintillator is ~ 32% for ^241^Am source (Table 1) which is 86% of EJ-212 detection efficiency ~ 37%. The thickness of epoxy scintillator is four time higher than that of EJ-212. Reduction is thickness can improve the pulse height amplitude of epoxy scintillator pulse height spectra. Preparing a scintillator of thickness less than 1 mm by casting is slightly difficult because of high viscosity of epoxy used here. However organic solvents can be used to reduce the epoxy’s viscosity making it suitable for casting ultrathin scintillator.

**Table 1:** Absolute detection efficiency for standard alpha, beta and gamma sources.

S. No	Source	Scintillator size	Activity (A (Bq))	Background count rate (N_B_ (cps))	Source count rate (N_A_(cps))	Absolute detection efficiency (ε)
1	^241^Am	Ø50 mm × 1 mm	3700	3.7	1188.3	32.01%
2	^90^Sr-^90^Y	Ø50 mm × 5 mm	5093	5.9	1824.3	35.70%
3	^137^Cs	Ø50 mm × 25 mm	48,760	29.4	3293.8	6.69%

#### Beta radiation detection

**Figure 6:**
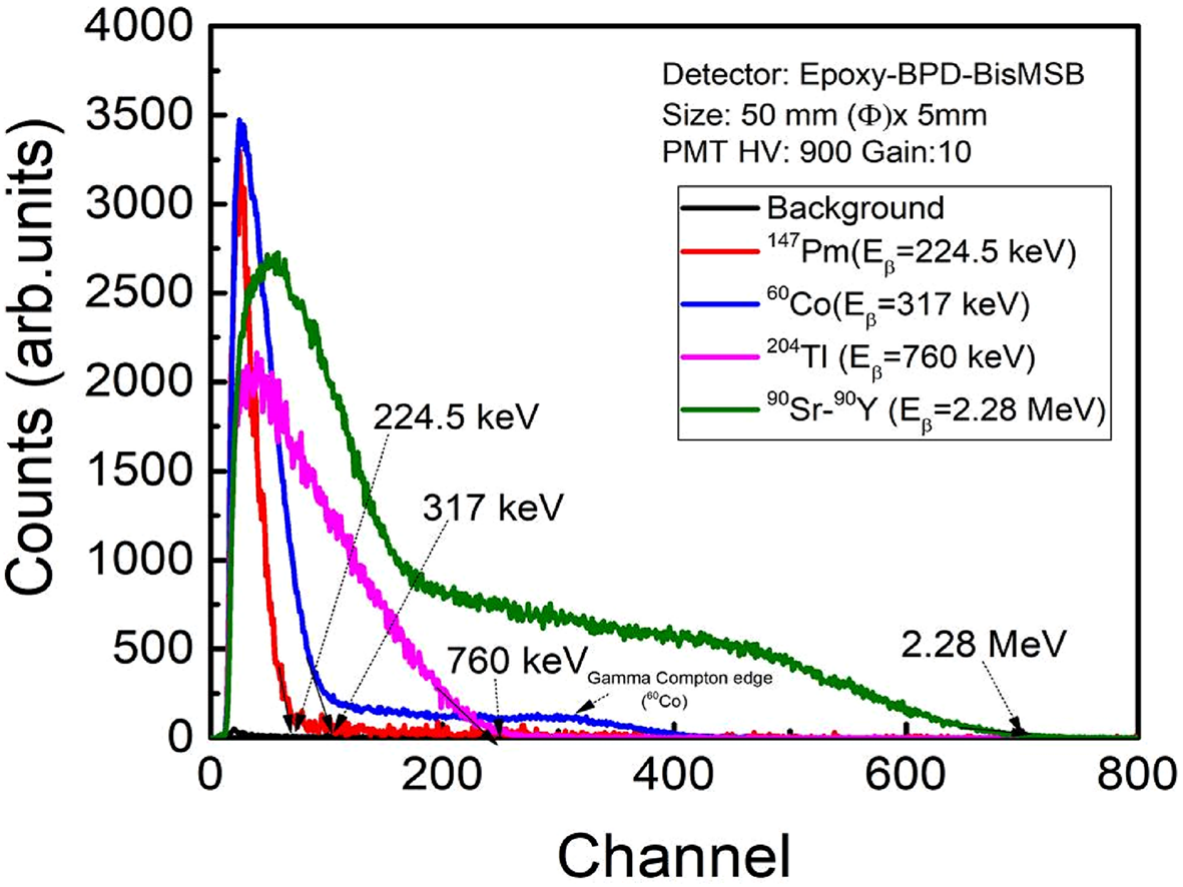
Pulse height spectra obtained using Ø50 mm × 5 mm scintillator detector with various beta sources (^147^Pm, ^60^Co, ^204^Tl and ^90^Sr-^90^Y).

**Figure 7:**
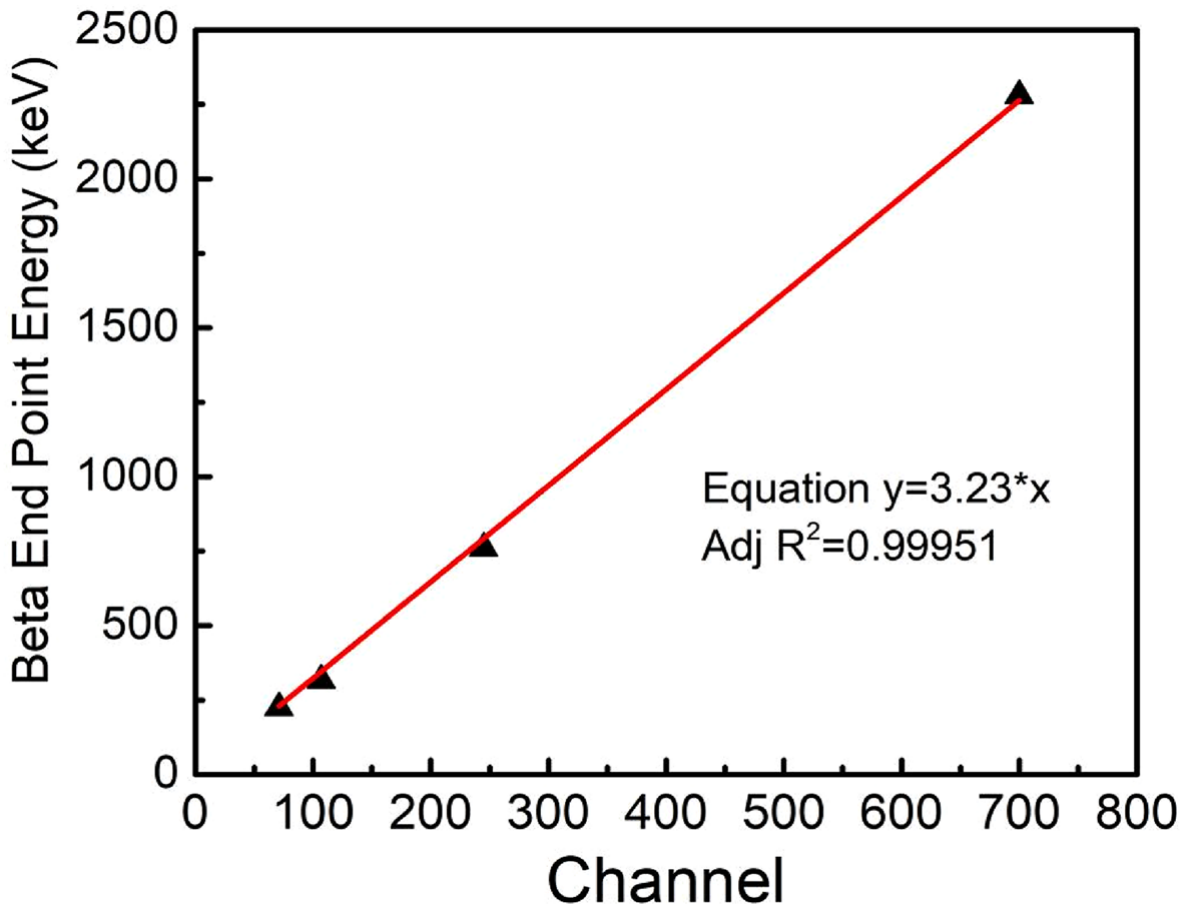
Beta end point energy of beta sources (^147^Pm, ^60^Co, ^204^Tl and ^90^Sr-^90^Y) plotted w.r.t channel no. corresponding to end point energy obtained from pulse height spectra.

Pulse height spectra acquired for duration of 300 s with Ø50 mm × 5 mm scintillator using different beta sources are shown in Fig. 6. For comparative view, spectra of low activity sources (^147^Pm, ^204^Tl) are enhanced to ten and three times respectively and that of high activity source (^60^Co) is reduced to half of originally acquired spectra. ^147^Pm pulse height spectra terminating at channel no. ~ 71, corresponds to end point energy (E_β_ = 224.5 keV). ^60^Co (E_β_ = 317 keV) has wider beta pulse height distribution and is terminating at channel no. ~ 107 along with distribution of low intensity pulses extending from channel no. 318 to 410 is observed. These pulses are due to Compton interaction of ^60^Co gamma radiation emitted post beta decay. As the beta end point energies are further increasing, the pulse height distributions get broadened. ^204^Tl (E_β_ = 760 keV) and ^90^Sr-^90^Y (E_β_ = 2.28 MeV) spectra terminate at channel no. 245 and 700 respectively. Unlike alpha particles, emitted beta particles are not mono-energetic but have distribution of energies with maximum possible energy as end point energy. Because of poly energetic nature of beta particles emitted from source, the pulse height spectra are broad and vary proportionally w.r.t. to end point energies of beta sources. Beta end point energies when plotted w.r.t. channel no. corresponding to beta end point energies, a straight line is obtained showing a relation between end point energy and channel no.. The slope of line ~ 3.23 keV/Channel is obtained as end point energy calibration factor (Fig. 7). The detection efficiency calculated for standard beta source ^90^Sr-^90^Y is found to be ~ 35.7% (Table 1). The effect of aluminium reflector foil on the beta energies was also studied. Energy loss of beta particles is calculated using ESTAR programme^37^ and tabulated in Table 2. Energy loss varies from 2.5% (^241^Pm) to 0.18% (^90^Sr-^90^Y).

**Table 2:** Calculated energy loss of beta particles due to aluminium reflector foil.

Sr. no	Beta source	Beta energy E_β_ (keV)	Mass stopping power (S) MeV- cm^2^/g	Linear stopping power L = (S*ρ_Al_)*1000 (keV/cm)	Energy loss in aluminium foil ΔE_β_ = L*t_Al_ (keV)	Percentage energy loss (ΔE_β_/E_β_)*100(%)
1	^147^Pm	224.5	2.072	5594.4	5.5944	2.49
2	^60^Co	317	1.814	4897.8	4.8978	1.54
3	^204^Tl	760	1.51	4077	4.077	0.54
4	^90^Sr-^90^Y	2280	1.535	4144.5	4.1445	0.18

#### Gamma radiation detection

**Figure 8:**
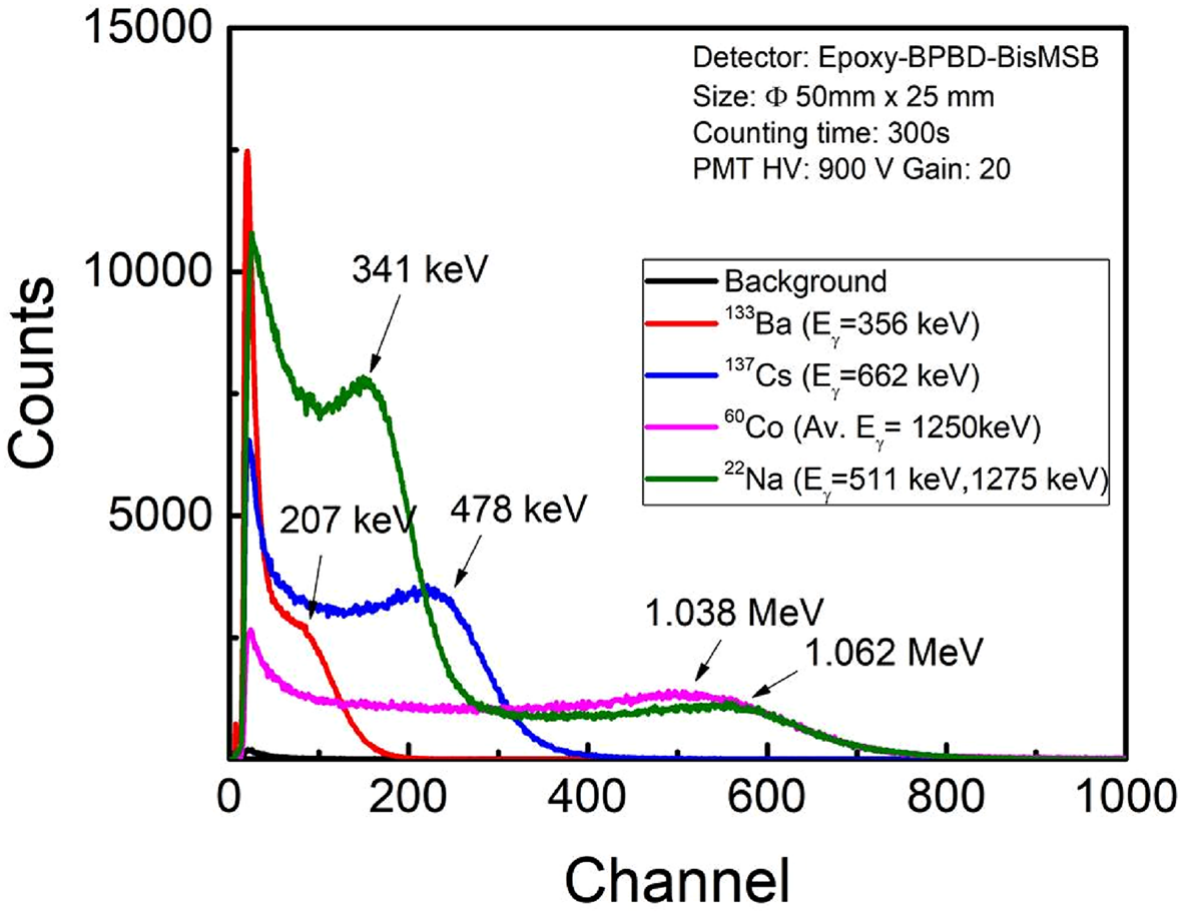
Pulse height spectra obtained with Ø50 mm × 25 mm scintillator using various gamma sources (^133^Ba, ^137^Cs, ^60^Co and ^22^Na). Calculated Compton edge energy is marked for each source spectrum.

**Figure 9:**
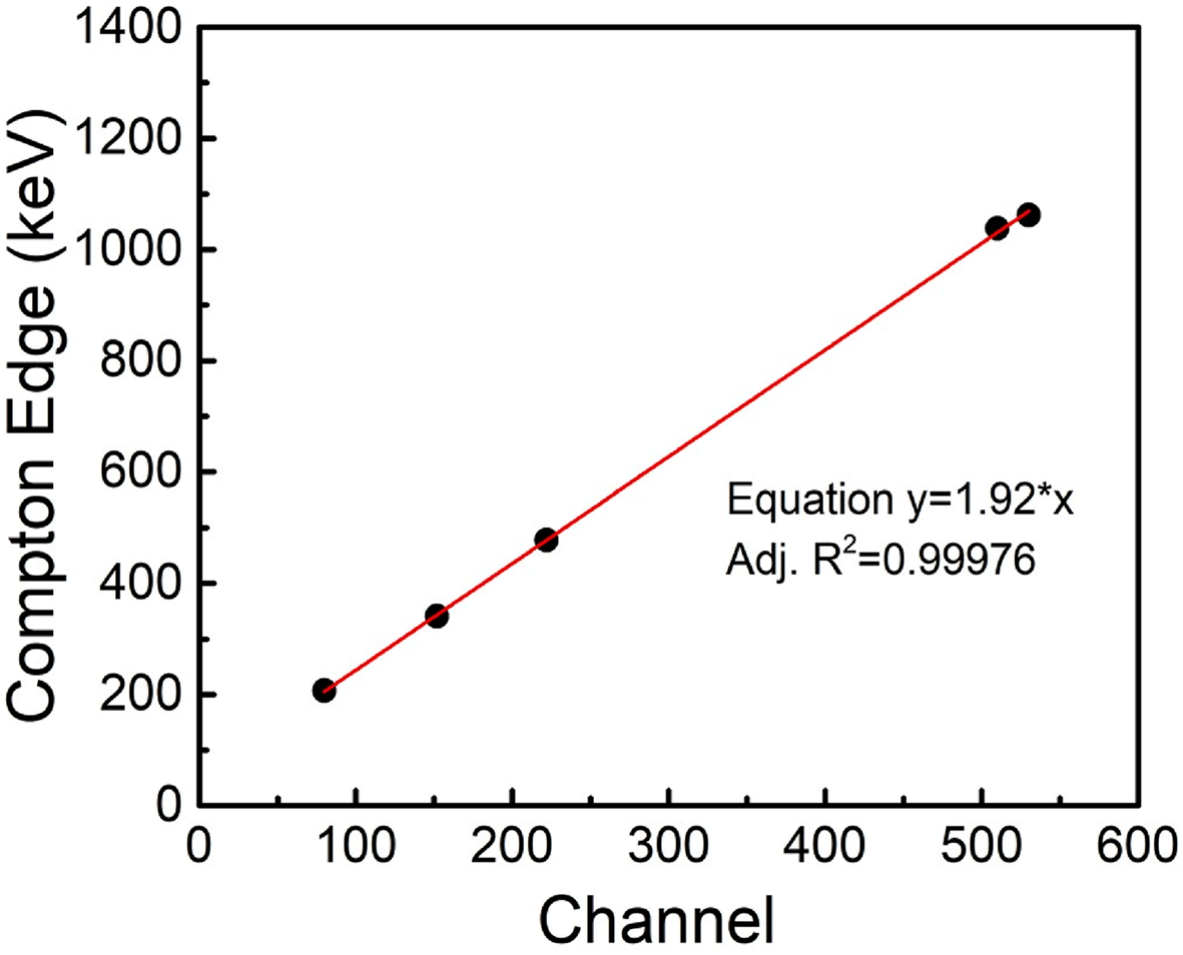
Compton edge energy of gamma sources (^133^Ba, ^137^Cs, ^60^Co and ^22^Na) plotted with respect to channel no. corresponding to Compton edge obtained from pulse height spectra.

Since epoxy’s (C_21_H_25_ClO_5_) effective atomic number is low (Z = 8.5) and gamma energies involved here are ranging from 356 to 1275 keV, Compton scattering is dominant mechanism of interaction due to high Compton cross-section^38^. The generated Compton electrons have range of energies depending on scattering angle of gamma photon from electrons in epoxy molecule. The maximum energy ${E}_{e}$ transferred to Compton electron by gamma photon of energy ${E}_{\gamma }$ is referred as ‘Compton edge’ which corresponds to gamma scattering at an angle of 180^0^. The Compton edge is given by equation

2 $${E}_{e}=\frac{2{(E}_{\gamma }/{m}_{0}{c}^{2})}{1+2{(E}_{\gamma }/{m}_{0}{c}^{2})}{E}_{\gamma },$$

where ${m}_{0}{c}^{2}$ is electron’s rest mass. The Compton electrons excite epoxy molecules which in- turn excite the fluorophore molecules, generating the scintillations. Figure 8 shows the pulse height spectra obtained with Ø50 mm × 25 mm scintillator using various gamma sources such as ^133^Ba (E_γ_ = 356 keV**)**, ^137^Cs (E_γ_ = 662 keV**)**, ^60^Co (Av.E_γ_ = 1250 keV) and ^22^Na (E_γ_ = 512 keV and 1275 keV).The pulse height spectra intensity of ^133^Ba is reduced to half for better comparative view. Gamma Compton edge is characterized by a hump in pulse height spectra, which is proportional to energy of gamma photon as per Eq. (2). Compton edges of respective sources are clearly visible in Fig. 8, confirming the dominant Compton interaction occurring in the scintillator. The counts obtained beyond Compton edge corresponds to multiple Compton interaction and photoelectric interaction which are relatively lesser intensity compared to that of Compton edge. The calculated Compton edge energy (Table 3) is marked for each source on the pulse height spectra in Fig. 8. Figure 9 shows plot of Compton edge energy with respect to corresponding channel numbers obtained from Fig. 8. The obtained straight line shows the relation between the channel no. and Compton edge energy and slope ~ 1.92 keV/channel gives the Compton energy calibration factor. The absolute detection efficiency is obtained to be ~ 6.7% for ^137^Cs (Table 1).

**Table 3:** Gamma energies of various gamma sources, their Compton edges and channel corresponding to Compton edges.

S. no	Gamma source	Energy photon (E_γ_ (keV))	Compton edge (E_e_, keV)	Channel corresponding to Compton edge
1	^133^Ba	356	207.2	80
2	^137^Cs	662	477.7	152
3	^60^Co	1170, 1330 (Av. ~ 1250)	1037.9	222
4	^22^Na	511, 1275	340.6, 1062.1	510

## Conclusion

Epoxy-bPBD-BisMSB composite plastic scintillator is prepared in different sizes using a cost effective solution mixing method for efficient detection of alpha, beta and gamma radiations. Radiation detectors were realized by coupling the prepared scintillators with PMT and MCA. Radiation response studies for alpha, beta and gamma radiations were performed on these detectors. Alpha pulse height spectra showed distinct peaks corresponding to different alpha energies with FWHM and detection efficiency comparable to a standard commercial scintillator EJ-212. Beta scintillator showed distinguishable beta spectra for different energies ranging from 224.5 keV to 2.28 MeV. The end point energies have linear variation with the corresponding channels, validating the spectroscopic measurement capability of the prepared detectors. Detection efficiencies ~ 32% (alpha) and ~ 35.7% (beta) enable these scintillators to be used for efficient detection of low level radioactive contaminations. Gamma spectra are broad due to low Z of epoxy base and ‘Compton edges’ obtained in pulse height spectra, validate the occurrence of dominant Compton interactions in scintillator. This Compton kinematics can be used for gamma spectroscopic applications. Also, gamma detection efficiency ~ 6.7% allows it to be used for radioactivity and dose rate measurements applications. The ease of preparation makes this suitable for large area scintillator fabrication without requirement of sophisticated infrastructure. Also these detectors combined with suitable converter material can be used for (n,α) and (n,γ) reaction based neutron detection.

## Data Availability

All data generated or analysed which support the finding of this study are available within the article.
